# Seemingly Harmless Differentiated Thyroid Carcinoma Presenting as Bone Metastasis

**DOI:** 10.1155/2018/8749075

**Published:** 2018-06-03

**Authors:** D. Magalhães, C. Costa, I. Furtado, M. J. Matos, A. P. Santos, H. Duarte, M. Afonso, J. Lobo, I. Torres

**Affiliations:** ^1^Endocrinology, Diabetes and Metabolism Department, Centro Hospitalar São João, Porto, Portugal; ^2^Faculty of Medicine of University of Porto, Porto, Portugal; ^3^Instituto de Investigação e Inovação em Saúde, University of Porto, Porto, Portugal; ^4^Endocrinology Department, Instituto Português de Oncologia, Porto, Portugal; ^5^Internal Medicine Department, Centro Hospitalar do Porto, Porto, Portugal; ^6^Nuclear Medicine Department, Instituto Português de Oncologia, Porto, Portugal; ^7^Pathology Department, Instituto Português de Oncologia, Porto, Portugal; ^8^Cancer Biology and Epigenetics Group, Research Center, Instituto Português de Oncologia, Porto, Portugal; ^9^Pathology and Molecular Immunology Department, Institute of Biomedical Sciences Abel Salazar (ICBAS), University of Porto, Porto, Portugal

## Abstract

Thyroid carcinoma is the most common endocrine neoplasia. Differentiated thyroid carcinomas (DTCs) represent the majority of cases, which usually follow an indolent clinical course with low mortality rates. The authors describe two cases of well DTC without classic histological poor prognosis features, presenting as extensive and unresectable osteolytic bone metastases. DTCs are considered harmless tumours, due to their benign and silent behaviour. The authors want to underline the importance of clinical awareness during follow-up in cases of DTC, which can be aggressive in presentation and behaviour. Timely identification and diagnosis of these tumours are essential for prompt treatment initiation and improvement of overall survival.

## 1. Introduction

The most common endocrine neoplasia is the thyroid carcinoma, with a rapidly increasing incidence worldwide [1–3]. The majority (>90%) of thyroid cancers are differentiated thyroid carcinomas (DTC)—papillary or follicular—which follow, in most cases, an indolent clinical course with low mortality rates [4, 5].

When distant metastases occur, the bone is the second most frequently affected site [6]. The incidence of bone metastases is about 1 to 7% in papillary carcinoma and 7 to 20% in follicular carcinoma. When present, the lesions are mostly osteolytic in nature [2, 7]. Bone metastases can result in clinically significant morbidity, coursing with important pain, pathological fractures, and neurological dysfunction [2, 6]. However, clinically silent presentation of bone metastases can also occur [2]. The presence of bone metastases is associated with a worse prognosis, lower survival rates, and significant deterioration of quality of life [2].

The authors report two cases of DTC without significant microscopic adverse features presenting as lytic bone metastases.

## 2. Case 1

**Figure 1:**
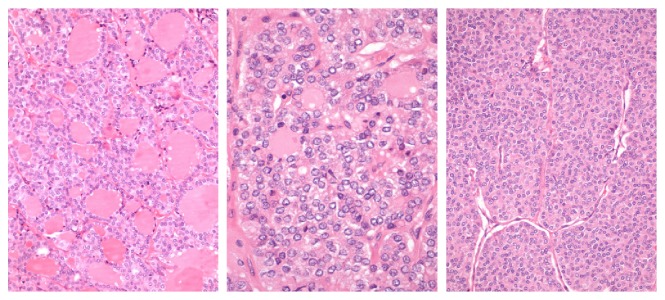
Fragments of bone tissue involved by epithelial neoplasia of follicular architecture, with foci of nondifferentiated (insular) carcinoma [Case 1].

**Figure 2:**
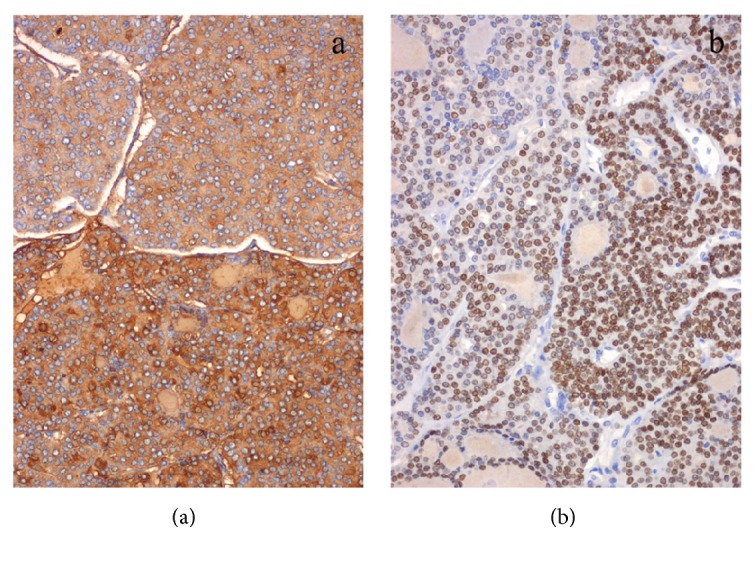
Malignant cells revealing intense and diffuse immunoexpression of thyroglobulin (cytoplasmic) (a) and TTF-1 (nuclear) (b) [Case 1].

**Figure 3:**
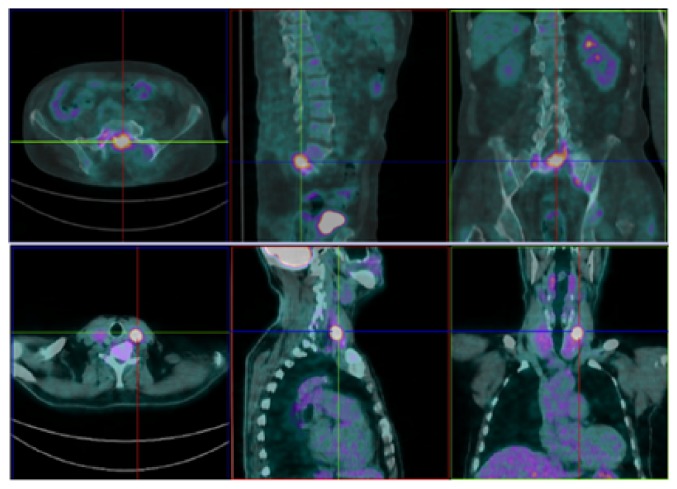
18F-FDG-PET showing uptake of the radiopharmaceutical drug in thyroid left lobe, L5 vertebrae, and pelvic bones [Case 1].

**Figure 4:**
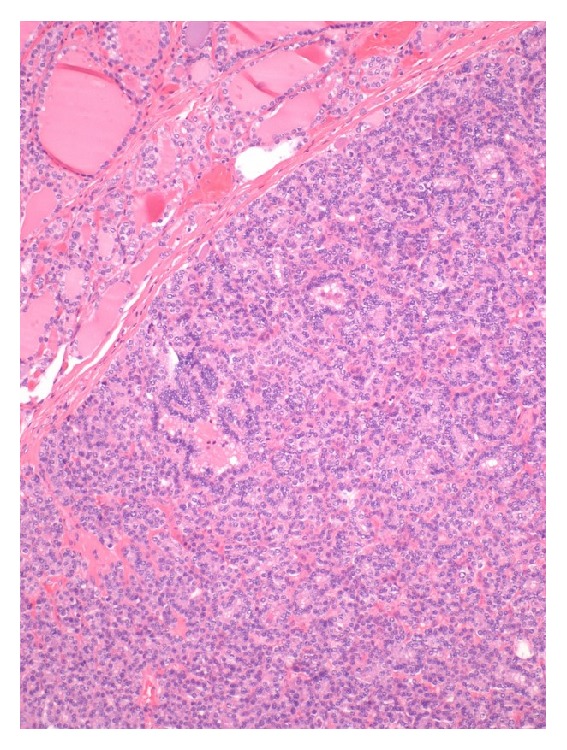
Fragments of thyroid involved by papillary thyroid carcinoma of follicular and oxyphilic variants [Case 1].

**Figure 5:**
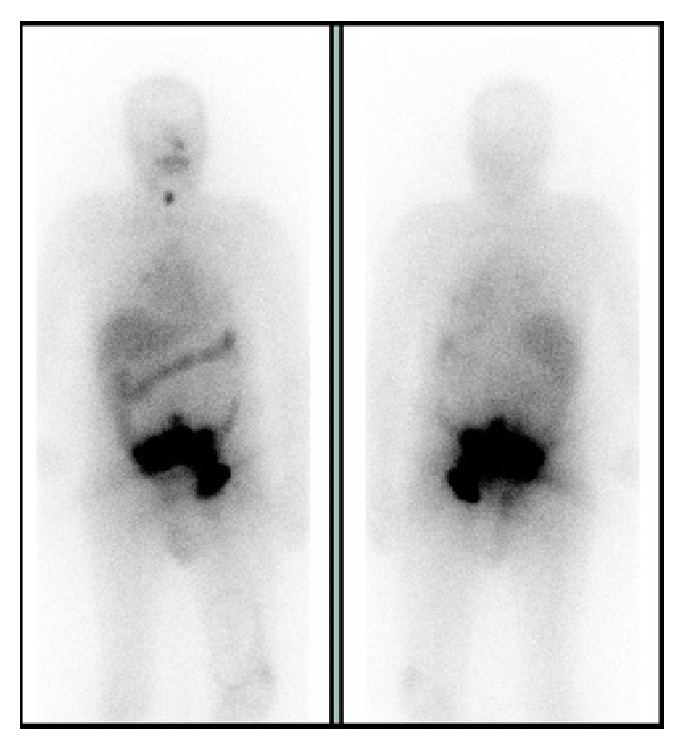
Posttherapy scintigraphy showing hyperfixation in the remnants of the sacrum and lower lumbar spine, bilateral iliac bone, and anterior cervical region [Case 1].

A 62-year-old male presented with refractory sacral coccygeal pain. The patient had past medical history of type 2 diabetes mellitus (treated with linagliptin/metformin), nontreated high blood pressure, right-sided hemiparesis following meningitis in childhood, nephrolithiasis, and smoking history. The pelvic computed tomography (CT) revealed a 9x7.5x9 cm bulky mass in the sacrum with locally increased soft tissue density, causing extensive lytic lesions of the sacred vertebrae and extending to the left iliac bone, suggestive of chordoma. The patient underwent total sacrectomy with partial excision and reconstruction of the left iliac bone. The anatomopathological examination revealed sacrococcygeal involvement by a thyroid carcinoma, as verified by immunohistochemical staining for thyroglobulin and TTF-1, predominantly papillary (follicular variant), however with foci of nondifferentiated (insular) carcinoma (Figures 1 and 2). Thyroid ultrasonography showed a solid nodule of 20 mm in the right lobe and two solid hypoechogenic nodules of 11 and 9 mm in the left lobe, the smallest with coarse calcifications. No lymphadenopathies were found. 18F-fluorodeoxyglucose positron-emission tomography (18F-FDG-PET) revealed a hypermetabolic focus in the left lobe of the thyroid, consistent with the suspected malignant neoplasia, and uptake of the radiopharmaceutical drug in the fifth lumbar vertebra and pelvic bones, consistent with secondary involvement (Figure 3). Consequently, the patient underwent total thyroidectomy. Histological examination revealed only follicular and oxyphilic variants of multifocal papillary carcinoma (at least six foci) and none of insular carcinoma, with dimensions ranging from 2 to 15 mm, without signs of hematogenic, lymphatic, or perineural permeation, as well as no signs of invasion of the capsule or extrathyroidal extension, with resection margins uninvolved by tumour (pT1b[m]NxM1R0) (Figure 4). Radioactive iodine therapy (RAI) was then performed. Posttherapy scintigraphy showed hyperfixation in the remnants of the sacrum and lower lumbar spine, bilateral iliac bone, and anterior cervical region (Figure 5). TSH-stimulated thyroglobulin was 24490 ng/mL. Follow-up magnetic resonance imaging (MRI) revealed persistence and progression of the pelvic lesion. At this time the patient was unable to walk, had a chronic indwelling bladder catheter, suffered from fecal incontinence, and presented with uncontrolled refractory chronic pain.

## 3. Case 2

**Figure 6:**
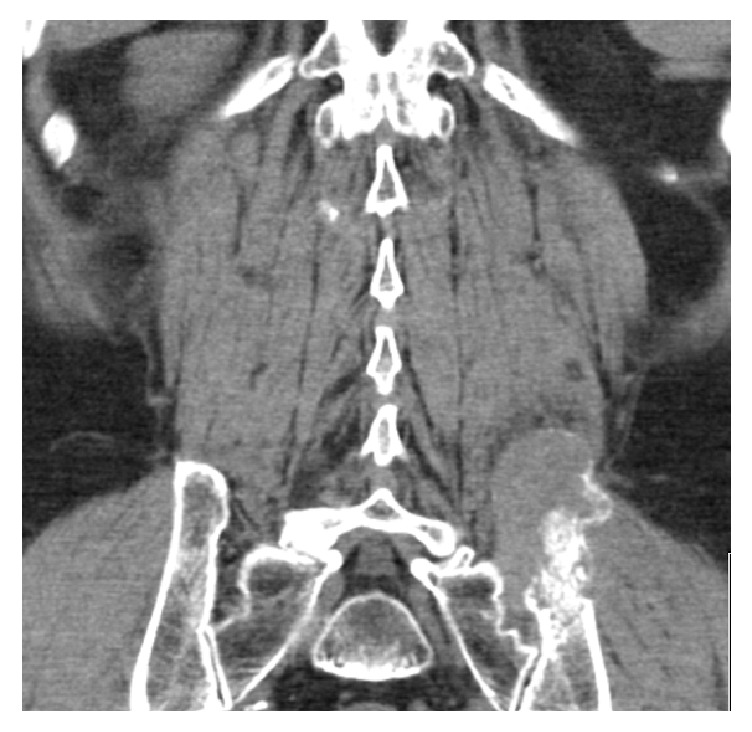
Pelvic CT revealing a 9 cm lytic lesion of left iliac bone [Case 2].

**Figure 7:**
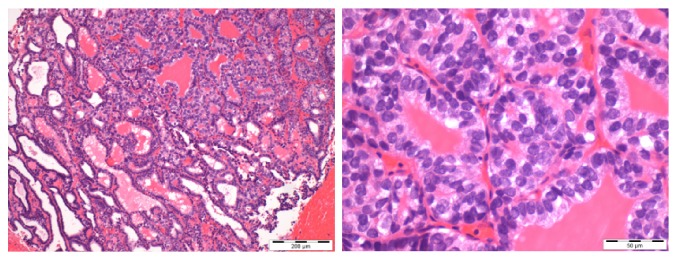
Fragments of bone tissue involved by epithelial neoplasia of follicular architecture [Case 2].

**Figure 8:**
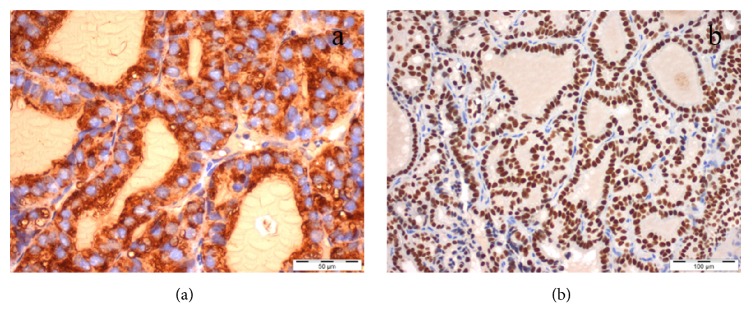
Malignant cells revealing intense and diffuse immunoexpression of thyroglobulin (cytoplasmic) (a) and TTF-1 (nuclear) (b) [Case 2].

**Figure 9:**
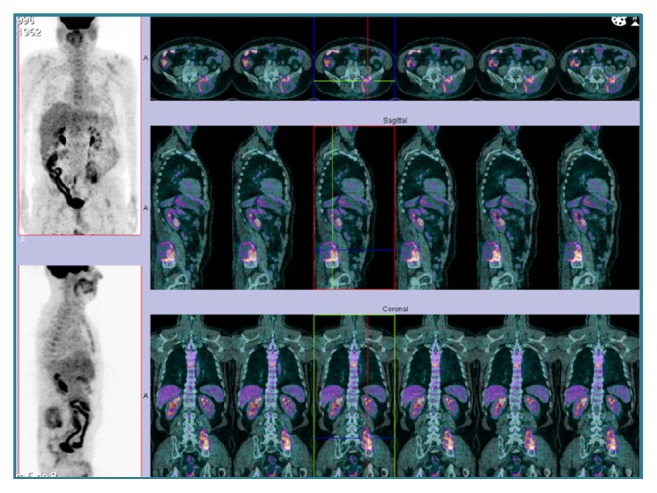
18F-FDG-PET revealing the pelvic lesion found on CT, without no other metabolically active lesions [Case 2].

A 64-year-old male presented with pain in the left iliac region for 6 months. His past medical history was significant for gastric peptic ulcer disease (submitted to partial gastrectomy and chronically treated with lansoprazole), nephrolithiasis, and hyperuricemia. At admission a poorly defined mass on the left posterior iliac crest was palpable. The patient underwent a pelvic CT, which revealed a 9 cm lytic lesion of left iliac bone with soft tissue involvement (Figure 6). A biopsy was performed and the histological examination and immunohistochemical staining for thyroglobulin and TTF-1 showed iliac involvement of a well-differentiated thyroid carcinoma (Figures 7 and 8). Thyroid ultrasonography disclosed a poorly defined 8 mm nodule in the left inferior lobe, heterogeneous and partially calcified, and a 4 mm hypoechoic nodule in the right lobe, without associated lymphadenopathies. The patient was submitted to total thyroidectomy and anatomopathological examination revealed a 1.1 cm papillary carcinoma, of follicular variant, with capsular invasion and limited extrathyroidal extension (ETE). Consequently, RAI therapy was performed. Postablative 131 iodine scintigraphy and 18F-FDG-PET (Figure 9) did not reveal further lesions. TSH-stimulated thyroglobulin was 185051 ng/mL. The patient is currently waiting for a hemipelvectomy.

## 4. Discussion

The authors present two rare cases of well DTC without significant histological poor prognosis features presenting as osteolytic bone metastases. DTCs, in particular those that are small and organ confined as in the above-mentioned cases, are often characterized by a slowly progressive course with a 5-year survival rate of 98.2% [8]. Of the several histological subtypes of papillary carcinoma, the follicular variants are probably the most common. At the time of diagnosis, distant metastases are seen in 2 to 10% of patients: two-thirds are pulmonary and one-fourth is represented by skeletal metastases [9]. Metastization at the time of the diagnosis of well DTC is an unusual combination, with poor prognosis. Moreover, according to the most recent data from Cancer Care Ontario, distant metastases occur in less than 1% of all thyroid cancer patients [10].

However, the bone is the third most common site of metastatic cancer of unknown origin, particularly of the spine, followed by the pelvis and long bones [11]. In such cases, bone biopsy is mandatory, since histological findings often provide important diagnostic clues, as well as important information regarding the prognosis and treatment of these patients [11]. This is particularly true in bone metastases from DTC [11]. In the first described case the radiological features were highly suggestive of a chordoma, dictating the choice of immediate resection, and pathological examination established the diagnosis of a thyroid carcinoma. In the second case, bone biopsy was diagnostic.

The described cases are unusual due to the aggressive presentation in the absence of previous poor prognosis anatomopathological features of the primitive tumours. In the first case report, beyond multifocality no other unfavourable features were present, predicting a low risk DTC [4]. However, the histological examination of the excised bone metastasis revealed insular carcinoma foci, suggesting primary tumour dedifferentiation during the process of distant metastization. In fact, DTCs can undergo dedifferentiation through a process of multiple genetic and epigenetic alterations, evolving into poor differentiated or even undifferentiated/anaplastic carcinomas [12]. The histological findings of bone metastasis in the first case, according to the Turin criteria, are compatible with a poorly differentiated thyroid carcinoma (PDTC), defined as a follicular-cell neoplasm with insular, solid, or trabecular growth [12, 13]. This type of tumour constitutes an entity that, in terms of morphology and behaviour, is considered of intermediate risk between papillary and follicular well DTCs and anaplastic carcinomas [12]. Significant regional differences in the incidence of this type of tumour are found, suggesting that their etiology is multifactorial and results from the interaction of genetic, environmental, and diet related factors [12, 14]. Furthermore, there are reports of tumours, which, even in the absence of any identifiable dedifferentiation marker, behave aggressively. In the second case report, capsular and microscopically immediate perithyroidal soft tissue invasion was present, estimating an intermediate risk DTC following 2015 ATA guidelines [4]. Nevertheless, the definition of limited/microscopic ETE is subjective, as the thyroid lacks a well-defined capsule and is often intermingled with adipose tissue or even peripheral skeletal muscle. Even so, recent studies have shown that gross ETE constitutes a strong predictor for recurrence and disease-related death, and that limited ETE alone does not have a significant impact on survival. Consequently, some authors questioned whether microscopic ETE should be sufficient to increase the risk of persistent/recurrent disease from low to intermediate or to upstage a DTC as T3 [1]. Furthermore, none of the cases presented with vascular or lymphoid invasion, which could predict a more aggressive conduct and the presence of extensive bone metastases.

Nevertheless, the factors predicting a worse outcome in DTCs also include demographic features. There is established evidence that male sex and increasing age are important risk modifiers [12]. The latest study from The Cancer Genome Atlas (TCGA) Research Network has shown that mutation density correlates with age and therefore age should always be considered as a continuous variable in risk stratification, rather than a static threshold from which patients are classified as having low or high risk [15]. The patients above-mentioned were both men and over 60 years old.

Although DTC has often an indolent course and usually poses minimal risk to human health, without apparent symptoms or adverse impact from their disease burden for many years, the cases mentioned above mean to underline the notion that patients can have an aggressive presentation and behaviour despite relatively “benign” histological features. In order to complement the histological study, it would be interesting to design a molecular study of the described tumours in an attempt to identify mutations that could serve as predictors for a more aggressive behaviour. DTCs are heterogeneous with different molecular signatures and variable presentation. A variety of molecular markers is involved in the processes of tumorigenesis, tumour progression, and development, including focal mutations, rearrangements, epigenetic changes of growth factors and their receptors, angiogenesis mediators, cellular regulators, and adhesion molecules. Because overall survival is improved by complete resection of the primary tumour and its metastases, early diagnosis and treatment are crucial.

## 5. Conclusions

DTC are commonly tumours with a benign clinical course. However, patients presenting with seemingly innocent features may have aggressive tumours with bone metastization upon diagnosis. Prompt treatment initiation is crucial for patient survival. The authors want to underline the necessity of high clinical awareness and close follow-up to decrease morbidity and mortality in these patients. Further studies are needed to disclose clinical and histological patterns of poor prognosis.

## Abbreviations

DTC: Differentiated thyroid carcinoma
RAI: Radioactive iodine therapy
ETE: Extrathyroidal extension.
